# Private Surgical Practice

**Published:** 1877-05

**Authors:** 


### PRIVATE SURGICAL PRACTICE.

Under the care of JOHN E. OWENS, M. D., Chicago.

### *Internal Hæmorrhoids; Operations by Clamp and Actual Cautery.*

Case 8. Mr. ——, about 50 years of age, came under my care in 1873. I had, at that time, never encountered a case where, in consequence of internal hæmorrhoids, the patient's life was in such danger. I am, at this time, unable to find my notes of the case, but the leading features I well remember. Having suffered for years, the usual symptoms, namely, protrusion, strangulation, inflammation, pain and hemorrhage, the patient had become exhausted to death's door. The prominent symptom was hemorrhage. Defecation became very inconvenient in the morning, in consequence of the loss of blood on such occasions, and its attending exhaustion. This matter was therefore postponed till evening, when injections accomplished what nature, unaided, might have done, at the proper time, in the morning. After a little experience, he soon found out that his strength was best conserved by going immediately to bed, and thus by the morning he had rallied sufficiently to ride down town, to his place of business. He did but little more, however, than sit in the office and give orders. In this way the patient dragged out a most miserable existence. The eyes and skin were of a greenish color, in consequence of which it was thought by many that the patient was laboring under some obscure disease of the liver, and for this he was treated at mineral springs and elsewhere. Prof. J. Adams Allen, his family physician, finally convinced him that hepatic disease did not exist. Suffice it to say that when I called, by appointment to operate, the patient was in a very critical condition. He was prostrate on his bed, with œdematous legs, feet and face, oppressed breathing, and very small frequent pulse. Dr. Allen feared the existence of pulmonary œdema. Of course the operation was postponed. Under rest and medical treatment he soon improved, and the pulse and respiration being good, I operated with clamp and actual cautery, June 1, 1873. Only two or three hæmorrhoidal tumors were found. The patient recovered without an untoward symptom, and became a useful man. I ceased surgical care of the patient, June 18. He is, at this date, March 21, 1877, in the enjoyment of health.

Case 9. Mr. C., aged 49 years, a carpenter, suffered in consequence of internal piles for twenty years. They bled more or less during the ten years previous to the operation, and having commonly protruded at stool, voluntarily returned with one exception when they became strangulated, and were replaced by his family physician, Prof. DeLaskie Miller. The hemorrhage frequently continued twenty minutes. Whilst at work the blood often saturated his clothing, and ran down into his shoes. In May, 1874, I operated with clamp and actual cautery. The patient made a good recovery, and was in the enjoyment of health, March 16, 1877.

Case 10. A lady, aged 59 years, from Ottawa, in this State, had internal hæmorrhoids for twenty-four years. Two of the tumors were uncommonly large. A "perineal" pile was, as is usual, particularly troublesome. Menstruation ceased twelve years ago. I operated with clamp and actual cautery, August 25, 1875. Suppression of urine immediately followed, and continued without inconvenience for 36 hours. Fomentation and sweet spirits of nitre were ordered. The urine remained scanty and high colored for two days; its passage was attended with considerable pain.

August 28th. Moderate protrusion of swollen mucous membrane; no pain in rectum. To this date a sufficient quantity of opium had been taken to ensure constipation.

August 30th. Confection of senna having failed to move the bowels, the following pill was ordered to be taken at bed-time: Podophyllin, ext. nucis vom.; ext. Belladonna, aa gr. $\frac{1}{4}$; ext. colocynth. comp. grs. j.

September 14th. Patient has been going to the table for three or four days, and is now able to go out. She remains quite well to this date, April 14, 1877.

Case 11. Rev. M., aged 41 years. This patient was referred to me for operation by his family physician, and gave the following history:

About twenty-one years ago the patient suffered from dyspepsia, induced, doubtless, by sedentary habits. He strained too much at stool, and almost daily during this period of twenty-one years, he was annoyed by a protrusion of internal piles. For twenty years, bleeding from the rectum was more or less frequent. Any intercurrent ill, like sore throat, "cold" in the head, etc., was sure to be accompanied by a rectal hemorrhage. Remaining long at stool caused a hemorrhage. By keeping the bowels in a lax condition, by rising promptly from stool, and putting the piles back, hemorrhage was, for the most part, prevented. Bleeding ceased when he assumed the recumbent position. He seldom lost four ounces of blood—two or three times a year—perhaps as much. This gentleman is very active and energetic, accomplishes an immense amount of work, has traveled around the world, and over our own country. He began to preach in 1858. The rule was, that after preaching he was obliged to hasten home, in order to replace the piles that had protruded whilst he was in the pulpit, and, grasped by the sphincter, caused an agony, which seemed, at times, unbearable. Often on his way home he was obliged to stop and support himself as best he could. They bled only at stool. When inflamed he was compelled to remain in bed for three or four days.

Oct. 9th, 1875. Assisted by Dr. C. M. Chamberlain, who is the gentleman's medical attendant, and Drs. Guerin and Simons, I operated with clamp and actual cautery. The sphincter was not over-distended.

Oct. 13th. Increase in the soreness and in the protrusion of the swollen mucous membrane—an unusual occurrence, since swellings and soreness do not increase after the third or fourth day, unless from some indiscretion on the part of the patient. The patient acknowledged, however, that the previous evening being unable to pass water, he got up and strained. Injection of warm castor oil at 10 o'clock, A. M., and in an hour afterwards an ounce of oil by the mouth. Patient is now as he always is, very nervous and fidgety. Cold applications were used locally. Opium was used to allay pain caused by a spasmodic contraction of the sphincter, and to keep the patient still.

Oct. 17th. Bowels acted several times in response to the oil, and operations were attended with some pain. Patient went down stairs just two weeks from the day of the operation.

Oct. 28th. Patient has been out daily since October 23d, and to-day made seven or eight calls.

March 15th, 1877. Patient is perfectly well at this date.

Remarks.—In a certain proportion of cases, suppression of urine without inconvenience to the patient, occurs. Retention of urine usually yields to hot applications, the use of the catheter being seldom required. Opium, perhaps, plays a certain part in the causation of the former, and yet in case 10 it was given very sparingly indeed. Brisk purgation is both unnecessary and hurtful. One or two operations on the third or fourth day, induced by the mildest means, are quite sufficient. Injections of warm castor oil, once or twice daily, using the first one, thirty-six hours before it is intended the bowels shall move, will in a proportion of cases be all sufficient. In all cases, whether an additional laxative is required or not, the oil injections add comfort to defecation. I have only on one occasion operated with the clamp and actual cautery, without the employment of an anæsthetic. The most painful part of the operation is the closing of the clamp on the pedicle. The application of the cautery was not complained of in the case referred to. The sphincter should always be over-distended, as the first step in the operation. This precaution having been observed as a rule, there will be but little complaint of subsequent pain. Three indications present themselves, namely, to lock up the bowels as long as may be necessary, to ensure quietude of the patient, to annul pain. To meet these, I give the preference to powdered opium. Cold water bathing for several weeks after the patient is able to get up should be recommended, and unless instructed, patients commonly do this in the most inefficient manner. If the patient will place a basin of cold water on the *floor* (not on a stool) and stoop down over it, bringing the anus as close to the water as may be, it will be found that the anus opens and the very lowest part of the rectum comes to a certain extent into view. The bathing is then best accomplished with the hand.

Formerly I *pulled* the piles down as is recommended in the books, and thus secured them one by one in the clamp. This method is objectionable. When traction is made upon the tumor the pedicle is elongated at the expense of the normal mucous membrane. The latter is thus drawn into the clamp. As soon, however, as the traction is relieved by the removal of the clamp, the margins of the wound are not only drawn more or less widely apart by the resiliency of the rectal walls, but the wounded surface is likely to bleed. The sphincter having been over-distended, the hæmorrhoidal tumors which come at once into view, should be, one by one, secured in the clamp at the pedicle before any traction whatever has been exerted upon them.

